# Energy-Efficient Crowdsensing of Human Mobility and Signal Levels in Cellular Networks

**DOI:** 10.3390/s150922060

**Published:** 2015-09-02

**Authors:** Paweł Foremski, Michał Gorawski, Krzysztof Grochla, Konrad Polys

**Affiliations:** Institute of Theoretical and Applied Informatics, Polish Academy of Sciences, ul. Baltycka 5, Gliwice 44-100, Poland; E-Mails: pjf@iitis.pl (P.F.); mgorawski@iitis.pl (M.G.); kpolys@iitis.pl (K.P.)

**Keywords:** crowdsensing, energy efficiency, mobility models, network modeling, mobility measurements

## Abstract

The paper presents a practical application of the crowdsensing idea to measure human mobility and signal coverage in cellular networks. Currently, virtually everyone is carrying a mobile phone, which may be used as a sensor to gather research data by measuring, e.g., human mobility and radio signal levels. However, many users are unwilling to participate in crowdsensing experiments. This work begins with the analysis of the barriers for engaging people in crowdsensing. A survey showed that people who agree to participate in crowdsensing expect a minimum impact on their battery lifetime and phone usage habits. To address these requirements, this paper proposes an application for measuring the location and signal strength data based on energy-efficient GPS tracking, which allows one to perform the measurements of human mobility and radio signal levels with minimum energy utilization and without any engagement of the user. The method described combines measurements from the accelerometer with effective management of the GPS to monitor the user mobility with the decrease in battery lifetime by approximately 20%. To show the applicability of the proposed platform, the sample results of signal level distribution and coverage maps gathered for an LTE network and representing human mobility are shown.

## 1. Introduction

Current mobile phones are becoming multi-purpose computers for which voice transmission is just one of the functions provided to the user. Smartphones are powerful measuring devices, equipped with multiple sensors and a large data storage capacity. They also allow one to wirelessly transmit the measurement results to any location on the Internet.

Smartphones are carried by the users to virtually any location they visit. Therefore, a smartphone seems to be a natural device for mobile measurements in order to realize the idea of crowdsensing. The mobile crowdsensing is a new sensing paradigm based on the power of various mobile devices. It is used to acquire local knowledge through sensor-enhanced mobile devices, where individuals use them to collectively share data and to extract information to measure and map phenomena of common interest [1]. Multiple applications have been proposed to use a mobile phone as a platform for the measurements and gathering of information about, for instance, human mobility, locations of interest [2], air quality [3] or wireless network connectivity [4].

Crowdsensing enables carrying on extensive measurements covering a large area with very limited costs, but requires user participation. Three obstacles to crowd scaling have been identified by Xiao *at al.* [5] using the analysis of papers that describe crowdsensing applications. According to [5], the barriers are: heterogeneity of the used hardware (the developed software needs to be available for the phone of the user willing to participate), the burden imposed on the users and bandwidth demands. The heterogeneity of the used hardware can be easily overcome by the implementation of the crowdsensing application on multiple types of devices, and the bandwidth demands can be minimized using compression or using WiFi transmission instead of cellular communication. Thus, the burden imposed on users becomes the most important limiting factor. In this work, we try to further analyze it by running a questionnaire among a group of 100 people and looking into more details of those limits.

We identified that people are willing to participate in a crowdsensing experiment only if the burden in terms of change in their behavior is minimal. We concentrated on using the smartphone as a platform for the sensing application. Our analysis showed that the burden can be interpreted as the requirement of interacting with the crowdsensing application (e.g., denoting the current activity) and the requirement of charging the phone more often. We show how to overcome those limitations by implementing an application for monitoring of the wireless signal level and human mobility. In crowdsensing, it is possible to minimize the burden imposed on the user by merging and performing on-line analysis of the data provided by multiple sensors available on the smartphone. Different sensors have different energy footprints: for example, the accelerometer requires very low power, while the GPS requires more power. By performing continuous analysis of the values provided by the low power sensors, such as the accelerometer, and the signal strength of the received cellular network, we were able to detect moments in which it is required to enable the high power sensors, such as the GPS. The proposed method can be easily extended to enable or disable other sensors, like the microphone to monitor the noise level. It also allows for identifying changes in human mobility to detect states, like walking, running, driving, *etc*., and correlating those states with other measurements. Below, we list the major contributions of our paper:

1. We present a novel platform for crowdsensing location and signal strength data (Section 4) and use it to collect preliminary data on human mobility and signal coverage in an LTE network (Section 6).
2. We improve the energy-efficient GPS tracking, for example by using signal strength of GPS satellites to avoid unnecessary battery drain, and show how to implement it for crowdsourcing (Section 5).
3. In an online survey of 100 people, we found that 37% of our respondents would agree to participate in crowdsourcing, but they expect a minimum impact on their battery lifetime and phone usage habits (Section 3).
4. By running a two-week experiment on various Android devices, we found that, on average, our tracking application reduced the battery lifetime by 20% (Section 5.3 pt 1).
5. By analyzing real data on 272,000 searches for a GPS fix (acquiring the radio signal from satellites to calculate the position) on mobile platforms, we found that 92% of searches stopped in under 30 s, and 84% of platform users experienced a daily GPS duty cycle below 5%, which roughly corresponds to one hour of movement a day (Section 5.3 pt 2).

The rest of the paper is organized as follows: in Section 2, we review the related literature; in Section 3, we analyze the barriers stopping people from participation in crowdsensing experiments; in Section 4, we describe a proposed energy-efficient crowdsensing measurement application; in the following section, we provide details regarding the GPS management to minimize the energy usage and describe the measured energy usage; we continue in Section 6 by showing sample results of six experiments carried out using the proposed platform to measure human mobility and to map LTE network signal coverage; Section 7 concludes the paper.

## 2. Related Work

The crowdsensing idea has been well defined and presented in [1] and in the recently-published book [6]. The survey in [7] shows that using mobile phones for sensing has a very large potential due to a big number of devices that can be used. During the last few years, a number of crowdsensing applications and platforms have been described in the literature, like OpenSignal [8], LifeMap, APISENSE—a platform providing a solution to build and deploy crowd-sensing applications for collecting experimental datasets [9] or MOSDEN—a scalable mobile collaborative platform for opportunistic sensing applications [10].

The problem of motivating people to participate in a crowdsensing experiment is presented in [11], with game theoretic analysis of human behavior and the proposition of an auction-based approach for incentives. Koutsopoulos [12] extends this approach, providing the calculation of optimal payment allocation that minimizes the cost of compensation provided for participants. However, the compensations are not always possible, and a large number of research applications of the crowdsourcing idea rely on people voluntarily helping in the research project [13] or being given non-financial incentives. The participants may be motivated by serious games, like Foursquare [14], or functions provided by the application only when the data are provided by the user (such as improved navigation accuracy [15]).

The amount of effort provided by a crowdsensing participant is another important factor influencing the willingness of participation in the research. The barriers have been discussed in [5] showing that people are more likely to help when the amount of effort required from them is minimal and when no extra cost, for example in the form of an additional data transfer, is imposed. The barriers limiting the application of crowdsensing in a business to business relation are discussed in [16]. The problem of scarce resources limiting the possibility of using the mobile phone for measurements is described in [17], with the battery capacity shown as the main factor. A similar problem is discussed in [18], where the authors propose an incentive mechanism for a mobile crowdsensing scheduling; however, the mechanism is only research carried out through simulations, and it is not implemented in a real environment.

The problem of energy awareness in crowdsensing has been analyzed recently in a few papers. In [19], a piggyback mechanism is proposed that collects the data when the sensors are used by an application. This approach significantly lowers the energy usage, but decreases the number of performed measurements and does not allow for tracking the device mobility fully. The energy-efficient location API described in [20] allows one to predict the location based on the assumption that most of the users repeat the same path every day, but it decreases the accuracy and may introduce significant errors in case the user does not follow his or her habits. Osin *et al.* [21] proposed a method to enable GPS for location measurement based on the 3D accelerometer, achieving energy savings of up to 27%. A similar method is used in [22], but with emphasis on the improvement of the location accuracy. In this paper, we further extend this approach to significantly decrease the energy usage, as is described in Section 4. Dong Zhao *et al.* [23] propose a novel model of opportunistic coverage, along with a measurement methodology to estimate the coverage quality and an adaptive sampling mechanism to improve energy efficiency. The collected user’s mobility traces are used to select which user should execute the sensing action. The method proposed in [23] minimizes the energy utilization by coordination between nodes on larger time scales, without detection of movement, while we concentrate on local optimization for each node in short time scales by the detection of changes in node location. Kjaergaard *et al.* in [24] present an energy-efficient system for tracking trajectory, in which the goal is to track straight segments of a path instead of individual coordinates. The paper describes a system built on the EnTracked position tracking algorithm [25], extending it to trajectory simplification and three new modes of operation: heading-aware (which employs the compass), distance-aware (which employs GPS data) and movement-aware (which employs the accelerometer). The authors show significant improvement in energy savings comparing to the EnTracked algorithm, while keeping the location error within the requested error bound. However, comparing to our work, the paper considers Nokia smartphones, assumes constant device orientation for the heading-aware mode and does not support the accelerometer while biking and driving. In our paper, we show a crowdsensing application that leverages GPS signal strengths and activity recognition for a more robust use of sensors commonly found on modern smartphones

In a recent series of works by H. Xiong *et al.* [26,27,28,29,30], the authors discuss the energy usage caused by data transfer in mobile crowdsensing systems. The papers describe methods that optimize participant selection in a crowdsensing experiment, so that the amount of redundant work is minimized while fully covering a geographical area of interest. The authors exploit an interesting idea of piggybacking the data over already established connections. By predicting the likeliness of voice calls in given times of interest, they distribute the tasks only to selected participants. The authors also propose the methods of minimizing the number of tasks allocated to users. Comparing to our work, we propose to provide each user with their full location history as a participation incentive; hence, we do not apply any selection mechanism, and the proposed crowdsensing platform provides slightly different functionality; we minimize the energy usage due to data transfer by using a single bulk transmission with the data of the whole day. However, the piggybacking technique may be combined with our proposal to further reduce the energy usage or to improve platform responsiveness by shorter upload cycles.

The energy minimization by selecting which user should enable sensing functions is also discussed in [31]. The authors propose the use of comprehensive sensing techniques in crowdsensing scenarios to select users according to a proposed novel algorithm to reduce the user contribution. The system is tested on real datasets with good results; however, there are no data of the testing system in a real-time running application. Furthermore, the nature of the BX Tracker application forces the user to run it constantly (if no data traces are to be complete), so as in above cases, such a mechanism is not needed. Kyon-mo Yang and Sung-Bae Cho in [32] develop a low power context-aware system using modular Bayesian networks. The proposed system uses a Bayesian network, altered using a Markov boundary. Such an approach reduces the energy usage and enables eliminating the GPS usage. Experiments, performed using five Samsung S5 smartphones used by students, gave promising results: the accuracy of GPS + 4 sensors was highest; however, using only four sensors without the GPS was only 5% less accurate, while consuming 50% less power. The method proposed in this paper gives better energy optimization, and the system described in [32] detects only context-aware information and does not collect mobility data, thus realizing a slightly different function.

The mobility measurement using crowdsensing is presented by Bin Guo *et al*. [33], who propose an interesting idea of cross-space public information reposting, tagging and sharing in their FlierMeet project. Although the project itself is not similar to our solution, the use of GPS while tagging is an interesting one. Potentially, the FlierMeet project can be a source of mobility traces. However, the authors do not mention anything about the energy-saving techniques they use.

## 3. Analysis of Barriers for Participation in Crowdsensing

To correctly identify the potential barriers limiting the willingness of users in taking a part in crowdsourcing experiments, we have carried out an online survey. It has been responded to by 100 users over a period of 10 days. We present the results below.

**Figure 1:**
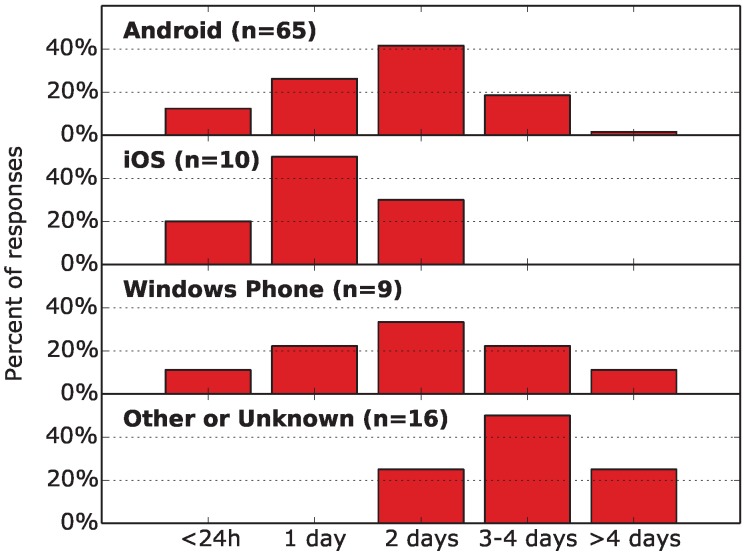
Single choice question: “In a typical daily usage, how long is your mobile phone able to work?”

**Figure 2:**
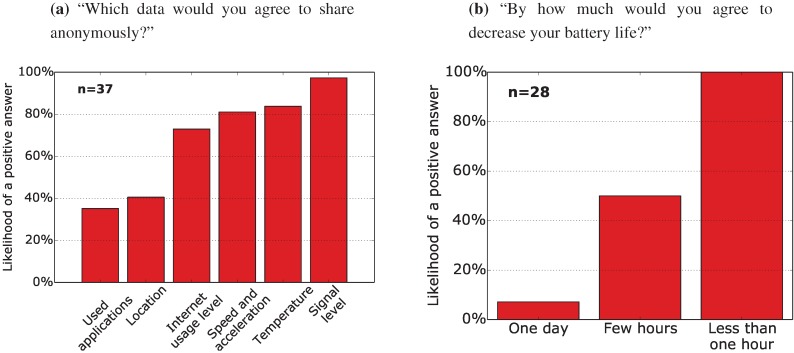
Multiple choice questions among: (**a**) respondents who agreed to participate in crowdsourcing; and (**b**) respondents who agreed to a shorter battery life due to data collection.

(1) Battery life: We asked the respondents about their battery lifetime during a typical daily usage, after the phone is fully charged. Figure 1 presents the results for different platforms. The battery lifetime differs, with the most popular answer of “2 days” for Android and Windows Phone, “1 day” for iPhone and “3–4 days” for other devices. The smallest battery life has been reported by iOS users: one day. The answers allow one to estimate the energy budget that a crowdsourcing application may use without influencing the phone charging habits of the users.

(2) Crowdsourcing opportunities: 37 respondents agreed to anonymously share their data for scientific purposes, which shows a significant potential for mobile crowdsourcing. Figure 2a presents the likeliness for sharing various types of data (a multiple choice question). As expected, people tend to object to collecting private data, like their location, which highlights the importance of incentives and proper data handling in such cases.

(3) Expected impact on battery life: Among the 37 respondents who agreed to participate in crowdsourcing, 76% (28 people) agreed to reduce their battery life due to the tracking application running in the background. However, people expect the battery impact to be as low as possible, preferably to reduce their battery lifetime by less than one hour. See Figure 2b for details.

(4) Expected impact on usage patterns: Among the 37 respondents, 51% (19 people) agreed to do additional tasks on their phones during the day, for example manually starting and stopping applications, entering data, *etc.*, but only 35% (13 people) agreed to do this once a day; and only one respondent agreed to do this more frequently. Generally, people expect no additional duties due to crowdsourcing or as little additional work as possible.

The results of the survey provide guidelines for the design of a mobile crowdsourcing platform, which should perpetually execute the measurements, with small battery usage and no additional tasks to perform during the day.

## 4. BX Tracker: An Energy-Efficient Crowdsensing Platform

To answer the need for real and accurate data that could be used to monitor the state of a cellular network or other measurements that can be executed using mobile phones, we have developed a novel BX Tracker platform. It was developed to minimize the barriers for participation identified in the previous section. BX Tracker is an energy-efficient crowdsensing application for Android smartphones and tablets. We designed it to accurately and perpetually track the user location and cell network characteristics without significant battery drain, which is an important factor for crowdsensing. Our platform also provides a web service that allows users to access their own data.

The main difficulty in the development of the application was the limitation of the energy used by the application. A crowdsensing measurements needs to be correlated with the location information. Currently-available tools, proposed in [4,9], execute measurements in constant intervals and acquire location information using, e.g., GPS for each measurements. This method is energy consuming, as the GPS needs to be enabled for each measurement. The novel approach implemented in the BX Tracker platform overcomes this limitation by constantly monitoring the accelerometer (which uses a few orders of magnitude less energy than GPS) and enabling the location measurements only when the user is moving. It allows limiting the amount of time at which the GPS is enabled, thus decreasing the overall energy usage.

Since deploying a test version in 2013, the system has tracked over 40,000 km of human movements from over 50 unique user identifiers. The collected data were successfully applied to modeling cellular networks: in [34], we proposed a novel human mobility model based on real data. Since September 2014, our mobile application has been publicly available on Google Play, under the name “BX Tracker” [35]. The system is now fully operational and used worldwide. We plan to convey experiments on cellular networks with a large user base.

Below, we describe the architecture and implementation of our platform and comment on the incentives for crowdsourcing location data.

### 4.1. Architecture

**Figure 3:**
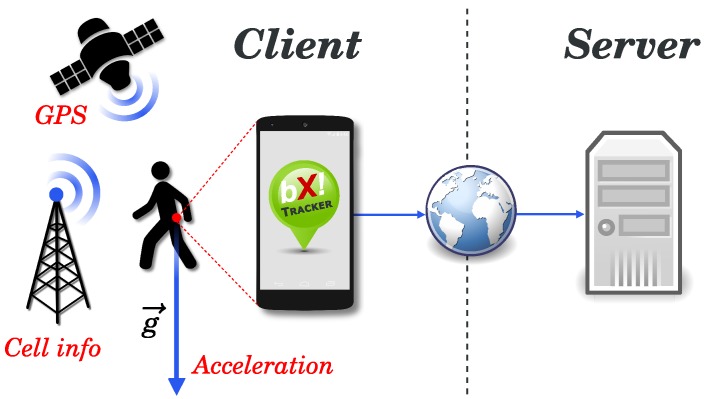
System overview. Pre-processed sensor data collected on mobile devices is periodically uploaded to the server.

The overview of our platform is depicted in Figure 3. The system is divided into two parts: the client side, where the BX Tracker application runs on possibly many Android devices, and the server side, where the collected data are processed and stored for later usage. The client application constantly acquires the data, applies basic pre-processing and filtering and uploads it to the server every 3 h over a secure Internet connection. The server immediately puts the uploaded data into a work queue that is later processed in a separate thread (it uses the producer-consumer scheme). Typically, the data will be available to the user a minute after the upload. In this step, the server converts raw data to KML (Keyhole Markup Languagage) files, computes the total distance traveled each day and extracts the names of visited cities. Finally, the data are stored in a database that is accessible to users through a web interface.

The reason for extracting additional information from the location data is two-fold: to attract the users with useful facts about their life and to help system operators quickly find interesting data, for example all users that traveled more than 1000 km last month. To collect the names of visited cities, we adopted machine learning and the Google Maps API. First, we apply the DBSCAN (Density-based spatial clustering of applications with noise) algorithm [36] to find clusters of geographical coordinates for a particular day, with parameters of $\varepsilon =5\times 10^{-3}$ and the minimum number of points equal to five. For each cluster centroid, plus the first and the last location of a given day, we query the Google Maps geocoding service [37] which converts geographical coordinates to street addresses. Finally, we select the most specific result and store the city name. To speed up the process, we also adopt caching for popular coordinates.

We ensure user privacy by assigning each user a completely random anonymous identifier (the “ID”) on the first run of the BX Tracker application. We adopted the UUID (Universally Unique Identifier) as the format for IDs, which makes them long enough for simple user authentication in our web service. However, the physical geographical coordinates are not anonymized. Note that proposing a dependable anonymization scheme even for coarse-level location data is problematic [38], especially if we want to retain the details of mobility patterns for each user. Thus, we store intact location data in the form that makes person identification as difficult as possible, that is we assign completely random identifiers to platform users. In such a setup, a sufficiently high number of users makes finding a particular person troublesome. Finally, having the original location data is essential for providing a useful web service.

### 4.2. Android Application

The client application runs on Android 2.3.3 (Gingerbread, released 2011) through 5.0 (LolliPop, released 2014), which constitutes about 99% of all Android installations [39] as of 2015. Android had almost an 85% share of the worldwide smartphone market in Q2 2014 [40], so we believe that BX Tracker is available for the vast majority of mobile devices sold nowadays.

The application was implemented in Java, using the Android SDK, as a “foreground service”, that is a Service process that constantly runs in the background and has a minimum chance of being killed by the operating system due to a limited amount of resources. Android OS saves battery power when it is able to turn the CPU off during inactivity periods; hence, we designed the service as an event handler instead of constantly checking for changes. Thus, at startup, the service registers for necessary events and then sleeps to conserve the battery power. We also provide the user with a graphical user interface (an Activity), which runs in a separate process and can be safely killed when needed.

**Figure 4:**
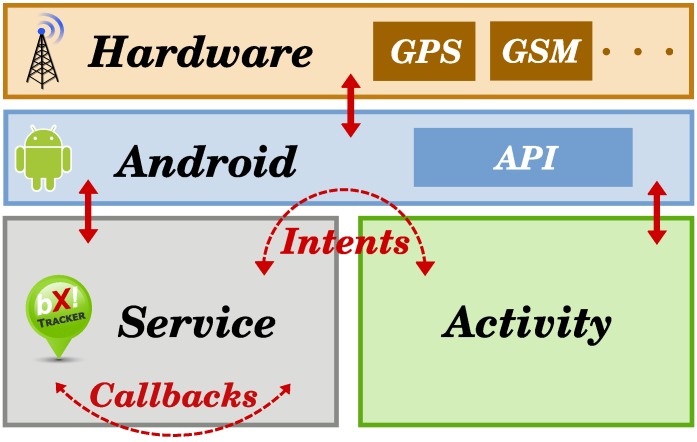
Architecture of the BX Tracker application.

The inter-process communication between the service and the user interface is realized using the Android Intent API, whereas the intra-process communication inside the service is realized using a custom callback system. The Android OS acts as a proxy between the application and the hardware and as a source of real-time clock interrupts. The BX Tracker architecture is briefly presented in Figure 4. The service part consists of many subsystems that will be described in detail in Section 5.

From the technical point of view, the most important feature in the BX Tracker application is user mobility monitoring. We rely on the location tracking subsystem that is able to work in a perpetual, accurate and unobtrusive manner, as will be explained in Section 5.

### 4.3. Collecting Data

Our platform can easily be modified to collect any data that needs geospatial features, like the popularity of public roads, frequent locations of voice calls or even city noise pollution [41], but we chose to measure human mobility and signal levels. See Table 1 for a listing of collected mobility data and Table 2 for signal data. Note that the data points of location and signal data do not always have the same values in the now column, because they are triggered by different events. Collected data are stored in CSV files named after the current date (e.g., 20140926-signal.txt), plus one text file with the details about the user device and the Android version (e.g., Sony D5503 4.4.4). On upload, all files are compressed as a ZIP archive and sent to the server over an HTTPS connection.

BX Tracker tracks location using only the GPS, because it is simple and universal. The collected data are stored in the columns lat, long, alt and acc. In addition to the GPS, the Android OS also provides the network-based location, and more recently, Google introduced its Fused Location Provider (FLP) [42]; however, these techniques depend on Wi-Fi for fine-grained results, which is not always available. Moreover, although FLP aims at fusing the best of the GPS with the network-based location, its high accuracy mode induces a 7.25% battery drain per hour, which effectively makes it infeasible for perpetual location tracking. Thus, we developed a new technique for GPS tracking, which is more universal than the network-based location and more energy-efficient than FLP (for our use case). However, we acknowledge that our technique described in Section 5 depends on a subsystem of FLP, Google Activity Recognition, which is used for the activity column. Note that the GPS works reliably only outside of buildings, but this is acceptable for our experiments: when the GPS signal is lost, the last known location will usually approximate the coordinates of the building in which the user is currently.

**Table 1:** Mobility data collected by BX Tracker.

Column Name	Description
now	Date and time of this data point (ISO 8601, UTC)
lat	Location: latitude
long	Location: longitude
alt	Location: altitude (m)
acc	Estimated location accuracy (m)
speed	Top speed in the last 2 min relative to now (km/h)
activity	Google Activity Recognition: in-vehicle, on-bicycle, on-foot, still, unknown
age	Time between measuring the location and now (s)

**Table 2:** Signal level data collected by BX Tracker.

Column Name	Description
now, lat, long, acc, age, speed, activity	The same as in Table 1
type	Cellular network type, e.g.: LTE, EDGE (Enhanced Data rates for GSM Evolution), HSPA (High Speed Packet Access), HSPA+
signal	Signal level (dBm)
netop	Mobile Country Code (MCC) and Mobile Network Code (MNC)
cid	Cell ID (CID) of the connected base transceiver station (BTS)
lac	Location Area Code (LAC)
signal-age	Time between measuring the signal level and now (s)

Mobile power saving techniques have a deep impact on the collected data. The Android OS aggressively tries to put the device into “deep sleep”, which can delay events, stop data collection for several hours or even completely remove the tracking process. As a work-around, we used a foreground service that runs in a dedicated process and uses the Android alarm manager for scheduling important tasks. Another important factor is the effect of the device screen being off. A popular power saving method is to disable all network interfaces in such situations. This could disrupt background monitoring if location tracking relied on Internet connectivity. For some rare hardware designs, the power source connected to the screen also drives the sensor chips, which makes the accelerometer unreliable on these devices. However, it is easy to detect such a situation and, thus, to mark collected data as faulty. Finally, our experiments showed that on many Android smartphones, the mobile signal level is not updated (or even reported) when the screen is off. Hence, by default, BX Tracker collects the signal data (Table 2) only when the screen is on; the location data (Table 1) are collected when the screen is either on or off. In case one needs fine-grained signal data, BX Tracker can periodically switch on the screen for a short period of time. On some devices, such a technique does not incur additional battery drain.

Unobtrusive and energy-efficient location tracking is the key requirement for mobile, location-aware crowdsensing systems that need perpetual user monitoring. We will show this in the next subsection. We highlight that our work can be generalized and used as a novel method in the field of crowdsourcing. The BX Tracker platform is an example of a successful application of our idea to measuring cellular networks on Android devices.

### 4.4. Attracting Users

In order to attract the users, we provide a free GPS tracking application on Google Play and a free web service available at [43].

**Figure 5:**
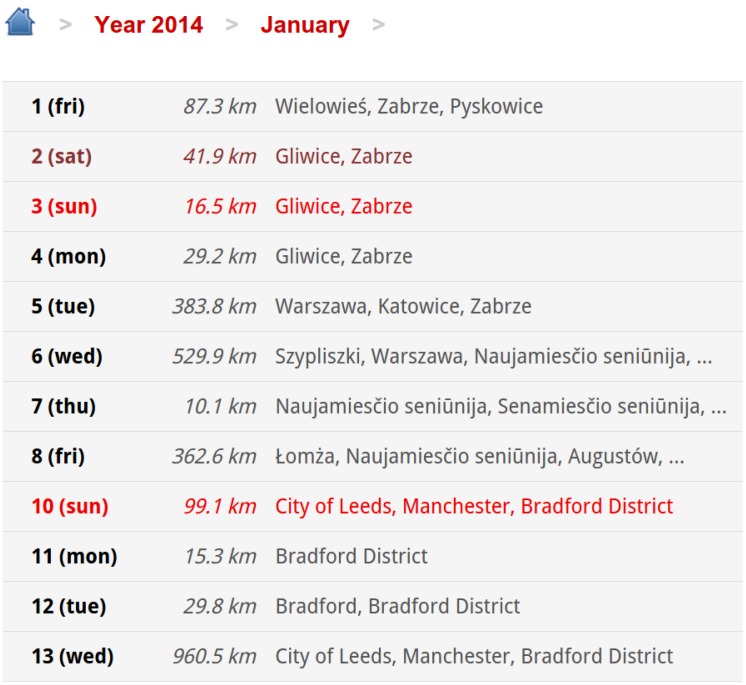
Exemplary screenshot of the web service. The image was cropped for clarity.

First of all, BX Tracker can be used as an ordinary GPS tracking tool for many tasks, like recording a walking tour. Compared to existing applications on the market, BX Tracker is designed to be energy-efficient by design; hence, the user does not need to control the tracking process manually. The application is free of charge and ads and runs on almost all Android platform versions.The web service provides the users with useful information on their travels. For example, Figure 5 presents an example of the month view: it lists days in a given month one by one, with information on the total distance traveled on a particular day and on the visited cities. The BX Tracker web service allows for storing the entire location history for years. Moreover, users can download KML files with their trips for each day and view the recorded routes online in a map browser, as presented in Figure 5. Finally, we provide the user with basic travel statistics: total monthly distance, weekday average and weekend average.

Many other incentives are possible for crowdsourcing systems that track location data. For example: geotagging photos taken with DSLR cameras, tracking time spent in various places or suggesting better options for everyday routes. The signal strength data can be used to pinpoint locations of base stations and places with a good signal for high-quality voice calls.

## 5. Energy-Efficient GPS Tracking

In this section, we show how to combine the GPS with the accelerometer to implement energy-efficient location tracking on mobile devices. We give a general idea using BX Tracker, which was designed for Android platforms, as an example.

### 5.1. Motivation and Idea

The original motivation for perpetual location tracking is human mobility modeling and location prediction, an important topic for cellular networks. Such research on human movements requires long-range, accurate and continuous location data of many individuals, which is a scarce resource. Some data are available in the CRAWDAD (Community Resource for Archiving Wireless Data At Dartmouth) database (for example [44,45,46]) and through the OpenStreeMap project [47], but none of the published datasets were suitable for our model presented in [34]. Moreover, we considered collecting the required data using off-the-shelf GPS trackers as inadequate. Apart from the costs, such an approach imposes additional duties on the volunteers and scales poorly. Thus, we decided to collect the data using mobile crowdsensing, which seems the natural choice nowadays [48].

Almost all new smartphones support location using various techniques. Apart from the traditional GPS location, smartphones offer network-based location, which employs wireless base stations as reference points. However, as already pointed out, such a technique is unreliable: in some regions, its accuracy can be as low as a few kilometers. On the other hand, popular GPS tracking applications are designed for short-term usage and, thus, require a manual start-stop procedure. As shown in Section 3, crowdsourcing volunteers expect minimal battery impact and no additional tasks to do. Hence, we propose a location tracking algorithm optimized for crowdsourcing data for modeling cellular networks.

The location can be tracked by having the GPS constantly on, but this could reduce the battery lifetime from 284 h down to 12 h [21]. On the other hand, people on average spend only an hour a day traveling [49]. Thus, our basic idea is to switch off the GPS chip whenever possible. In order to do that, we monitor the phone accelerometer for various activities, observe the signal strengths of GPS satellites, dynamically adjust the fix interval and track the user speed history.

In [50], Galeana-Zapién *et al.* proposed to solve the energy saving problem with a middleware architecture and a runtime environment that directly interfaces with application programming interfaces and device sensors, to manage the duty cycle process based on energy and context aspect. In comparison to that work, our solution optimizes energy usage and generates mobility and signal strength data in a single Android application. Moreover, we use the accelerometer to manage the GPS and granularity of measurements, and we monitor the signal strengths of GPS satellites to switch GPS off inside buildings. These techniques assure an energy-efficient usage of location sensors without third party optimizers.

### 5.2. Implementation

**Figure 6:**
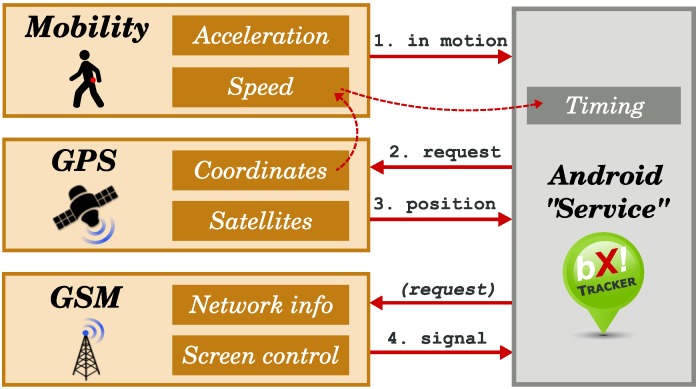
Implementation of the BX Tracker application.

A more detailed architecture of BX Tracker is presented in Figure 6. The system is divided into subsystems responsible for tracking various phenomena (visible on the left-hand side), and the Android Service part that controls these subsystems and receives callbacks (right-hand side; see Section 4.2). Note that the GSM subsystem, which collects signal quality data, is optional with respect to the overall system architecture. It can be replaced with another data collection subsystem, as explained in Section 4.3, or omitted. In Section 6, we show practical applications for the additional data. Below, we describe the crucial processes implemented in our location tracking method.

(1) Mobility tracking: Let us start with an observation that people have long periods of time during the day in which they do not change their location, for example: sleeping, staying at home, working at the office [48]. It is unnecessary to periodically check for the GPS position when no significant movement is recorded by the smartphone sensors. Another observation is that once people start moving, we can monitor the maximum speed attained in the last few minutes to ensure that they are still mobile. Thus, it is unnecessary to check the sensors until people stop moving for some time. We used these two simple observations as the motivation to build the Mobility subsystem (see Figure 6): it aims at detecting whether the user is currently more likely to be in motion or to be stationary. When the user is in motion, we request a new GPS fix. However, we also obey a minimum time pause between successive requests, as will be explained later.

The physical activity of a smartphone user can easily be detected using an accelerometer. This is a well-known research topic [51,52,53,54,55,56], so we adopted an off-the-shelf solution available in the Google Play Services software library, v. 6.1: Google Activity Recognition [42]. Once enabled, it will periodically send notifications about the detected activity [57] “in vehicle”, “on bicycle”, “on foot”, “running”, “walking”, “tilting” and “still”. The expected period between the updates can be configured, and each notification contains a confidence level. In BX Tracker, we set the interval to 10 s, and we skip the updates with the confidence level below 54%, which was verified experimentally to give the best results. Finally, when we receive a certain activity update of “tilting” or “still”, we consider the user to be stationary. We map the other classes to simple representations: see activity in Table 1.

The mobility tracking subsystem also passively listens to GPS location updates. Whenever the user moves more than 35 m in less than 2 min (about 1 km/h), the instantaneous speed and the distance between the two consecutive updates are recorded in a circular buffer with a timestamp value. The buffer is later used to retrieve the maximum speed and the maximum distance in the last 2 min, relative to the query time. If the speed is above 4.5 km/h or the distance is larger than 200 m, the user is considered to be in motion. Note that if no new GPS positions are received, the speed and distance data will be invalidated with time.

(2) GPS control: The task of the GPS subsystem is to handle requests for a GPS fix in an battery-efficient manner. We observed that it is often unnecessary to leave the GPS active for periods longer than 30 s. Such an amount of time is long enough to discern between a meaningful position search that is going to succeed (e.g., good GPS signal, outside of a building) and a process that is finally going to fail (e.g., GPS started inside an office room). Data collected from users confirmed this: in Section 5.3 pt 2, we analyze the time our algorithm needed to stop the GPS. The reason could be either obtaining a valid fix or observing a poor signal. For the vast majority of examined cases, the GPS was stopped in less than 20 s, with almost all fix attempts finishing in less than 1 min.

Based on the observations above, we implemented a GPS status monitor. Whenever we request a GPS position fix from the Android OS, we also register for updates on the GPS satellite signal strengths, which should be available each second. For each signal strength report, we calculate a “GPS score”: *S*, a metric that quantitatively estimates the quality of the GPS signal at the current location:

(1) $$\begin{array}{cc} S= & \max X+\frac{1}{2}\left|X\right|\\ X= & \left\{x_{i}:x_{i}\geq 10\right\} \end{array}$$

where $x_{i}$ are the signal-to-noise ratios of the GPS satellites [58]. We experimentally obtained a threshold value for *S*: if the maximum *S* value for the last 15 s was below 30.5, the fix is likely to fail, and thus, the GPS chip can safely be shut down to conserve the battery power. On the contrary, if this value is above the threshold after 30 s since the GPS start, we let the process continue, and we recheck it every 15 s. Finally, the GPS is unconditionally stopped after 5 min, which was verified experimentally to work well. As a work-around for Android-specific issues with the satellite signal reporting, we restart the GPS fix and the GPS reporting processes every minute, with no impact on the presented procedure.

We also implemented simple filtering and processing of GPS results. On a new GPS fix, if the last successful fix was more than 5 min ago, we ignore the first two results and let the GPS run. This was verified experimentally to reduce the noise in GPS paths on several smartphones. For the same reason, we also ignore the results with an accuracy worse than 100 m, with at most 10 tries to obtain a higher-quality result. If the result passes the presented filtering procedure, we stop the GPS and store the new location. We also calculate the instantaneous speed and the traveled distance, as mentioned in the previous point.

(3) Proper timing: Let us make another observation that different types of mobility require different time pauses between successive requests for a GPS fix. For example, if our requirement is 100-m accuracy and the user walks, the pause can be around 30 s, because it is unlikely that the user will be able to walk more than 100 m in such an amount of time. On the other hand, if the user drives a car in a city center, the pause time should be around 10 s or less, assuming an average speed of 36 km/h. Finally, if the user drives a car on a highway, we can increase the pause time, as highways are designed to avoid sharp road curves. The GPS track on a highway can be approximated with a straight line connecting two distant positions.

The Service part of the system presented in Figure 6 is responsible for imposing a pause regime on the GPS subsystem, using the information on user activity and speed. When the Mobility subsystem reports that the user is mobile, a GPS fix request will be generated only if a sufficient amount of time, *d*, has passed since the last successful fix. See Table 3 for the pause times used in BX Tracker, which were derived experimentally.

**Table 3:** Dependence of GPS pause time on user activity and speed.

Activity	*d* (s)
in-vehicle	15 $\;v>60\frac{km}{h}$
	10
on-bicycle	20
on-foot	30
Otherwise	60 $\;v<6\frac{km}{h}$
	10 $\;v>60\frac{km}{h}$
	360/v

(4) Collecting additional data: If possible, the event of a new GPS location should trigger collecting additional data; in the case of BX Tracker, the data about the mobile signal strength and network information. In such a case, an error in the geographical position is minimized, and the geospatial features of the measured phenomena are accurate. However, as previously noted, the power saving functionality of smartphones can disable some of the phone features when the device screen is off, which can be a common situation when the user is in motion. Hence, the subsystem responsible for recording additional data can either turn on the screen for a short period of time or fall back on opportunistic data collection, in which the data are stored only if the screen was on at the time of measurement. In BX Tracker, the user can manually choose between active and passive mobile data collection methods. In active mode, the Service subsystem will request turning on the screen and measure the signal data each time the user moves to a new location, plus an additional measurement will be made each minute disregarding user mobility. Collecting the mobile network signal is one of the additional BX Tracker features that is meaningful for both practical and scientific use. This mechanism for sampling the network signal is used in the BX Network application [59]. This application automatically samples both the Wi-Fi network (if accessible) and the mobile network and switches them according to network efficiency. The data collected from BX Tracker was used in [60,61], where the authors research the characteristics of signal strength changes in buildings and the possibilities of simulating this phenomena.

### 5.3. Battery Impact

In order to verify the effectiveness of our battery saving method, we evaluated BX Tracker on real mobile devices. Our goal was to learn how the perpetual location tracking affects the battery life and how it aligns with user expectations. We ran an experiment with a group of volunteers, and we analyzed the real data stored in our BX Tracker platform.

(1) Two-week experiment: First, we measured battery usage on 12 real devices carried everyday by a group of 10 people. We asked them to install a battery monitoring application and to use their devices in a typical way for one week, without BX Tracker. Then, we asked our volunteers to install and run BX Tracker for one more week, while still monitoring the battery usage. We also asked the group not to change their phone usage habits during the whole experiment. Of course, we acknowledge that we could not fully control all of the factors that impact the battery usage and that our sample group was small. Hence, the obtained results only approximate the real battery impact of BX Tracker, averaged for different mobile devices.

In order to monitor the battery usage, we provided the experiment group with a custom application. Whenever a phone charger was connected to the device, the application logged information about the percentage of the battery that was used, relative to the most recent event of the phone charger being disconnected. In other words, we monitored the amount of energy that was used after each time the battery was re-charged. We also logged the date and time of measurement, the time since the last re-charging and the information if the BX Tracker service process was running. We filtered the data by ignoring the measurements for which: (i) the user re-connected the charger quicker than after 2.5 h; (ii) the user re-charged the battery in the morning after <10 h, that is after sleep; and (iii) the battery level decreased by less than 5%. After two weeks, we collected the information and analyzed the change in battery usage for each device separately. For four devices, the logged data did not indicate that the BX Tracker process was running or we received very few measurements (1–2), so we skipped them. For the rest of the eight devices, please see Table 4 for the obtained results.

**Table 4:** Impact of BX Tracker on battery consumption, as measured on various mobile devices. BD: average battery drain per hour; BL: estimated battery lifetime on a full charge; E: estimated energy usage.

Device	Battery (mAh)	No Tracking		Tracking		Difference		
		BD (%/h)	BL (h)	BD (%/h)	BL (h)	BD (%/h)	BL (h)	E (mWh)
Galaxy S2 Lite	1500	2.4	42	3.9	26	+1.5	−38%	+83
Galaxy S3	2100	4.2	24	5.0	20	+0.8	−16%	+62
Galaxy S4	2600	3.5	29	5.3	19	+1.8	−34%	+170
Xperia Z1 C	2300	1.9	53	2.4	42	+0.5	−21%	+43
Xperia Z2	3200	1.4	71	1.5	67	+0.1	−7%	+12
Nexus 5	2300	3.2	31	3.7	27	+0.5	−14%	+43
ME302KL No. 1	6760	0.6	167	0.7	140	+0.1	−16%	+28
ME302KL No. 2	6760	0.7	152	0.8	130	+0.1	−15%	+30
*Average:*						+0.7	−20%	+59

The first six devices are the popular smartphones made by Samsung, Sony and LG, whereas the last two devices are Asus tablet devices, with a much larger battery capacity. We consulted manufacturer data to obtain the declared battery capacity and assumed that a 100% battery level corresponds to the declared values. The BD metric presented in the table measures the average battery drain per hour, and the BL metric estimates the battery lifetime on a full charge, given the BD value: BL=100/BD. The E metric roughly estimates the real energy usage, assuming the average battery voltage of 3.7 V, which is common: E=BD/100·3.7C, where *C* is battery capacity.

As shown in the table, BX Tracker on average decreased the battery lifetime by 20%. For the most popular battery lifetime in our survey, two days, this corresponds to decreasing the lifetime from 48 h down to 38 h. This still could be enough to give the user an acceptable phone experience, but in our survey, the respondents expected a decrease in battery lifetime by less than one hour or less than a few hours (see Figure 2b). However, our results show that even with aggressive energy optimizations, it is difficult to completely meet user expectations. From the technical point of view, the results show that it is possible to perpetually track the GPS position on an Android smartphone without draining the battery in a dozen of hours. We also estimated the real energy usage to enable comparison between smartphones and tablets, which differ in the battery capacity and, thus, have a different battery drain per hour. BX Tracker on average used 59 mWh, with a possible outlier (the Galaxy S4), which was probably caused by some other applications being activated on the phone in the second week of our experiment.

**Figure 7:**
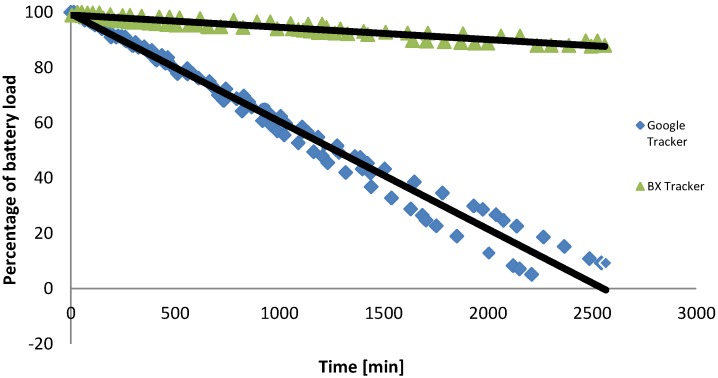
Comparison of battery use for BX Tracker and Google Tracker.

The next experiment compared the energy utilization of the proposed BX Tracker application to a popular location tracking application: Google Tracker. The tests were performed on two identical Asus ME302KL tablets, both with the same software, not connected to the Internet and not running any additional applications in the background (the difference in their discharge characteristics without tracking application was lower than 1%. Google Tracker was installed on one device and BX Tracker on the other device. The devices were turned on only during travel and keeping them always on with active trackers. The cumulative results of the test repeated twice are shown on Figure 7. After 2500 min of tests, the BX Power used less than 20% of the battery capacity, while the device running Google Tracker has been completely discharged.

**Figure 8:**
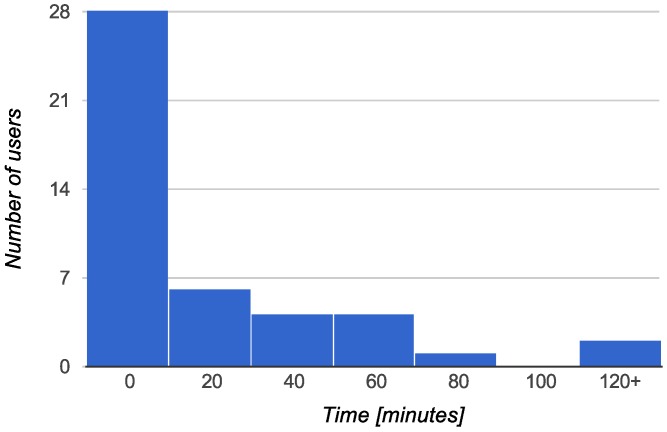
Total time of GPS activity per day. Measurements on 44 unique user identifiers.

**Figure 9:**
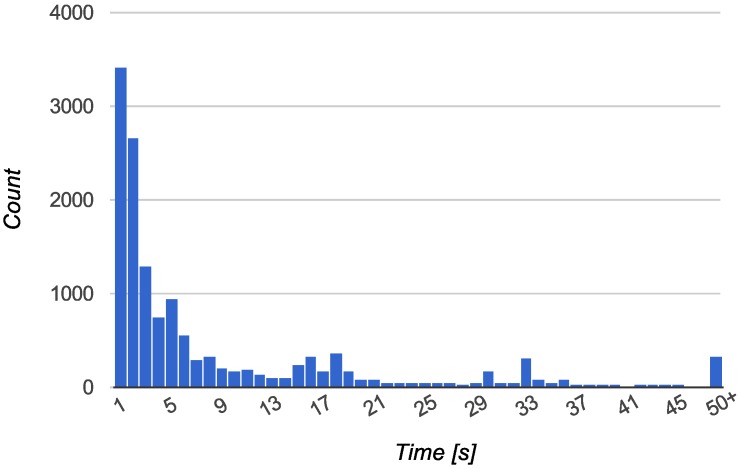
Time of GPS activity before providing a position fix or being disabled due to poor signal. Random 5% sample from 272,000 measurements on 44 unique user identifiers.

(2) Collected data: We also analyzed the real data stored in the BX Tracker database, collected for one year by 44 different user identifiers. The data corresponds to over 272,000 events of searching for a GPS fix. We analyzed two factors for the tracked devices: the daily duty cycle of the GPS chip, presented in Figure 8, and the time between starting and stopping the GPS fix procedure, presented in Figure 9.

For the data presented in Figure 8, the majority of users experienced the total time of the GPS chip being on for less than 20 min in 24 h, which corresponds to a 1.4% duty cycle. Moreover, 84% of users experienced a duty cycle of 4.2% (60 min) or less, and 95% of users experienced a duty cycle of 8.3% (2 h) or less. The average total daily activity of the GPS was 29 min (2.0% duty cycle).

For the data presented in Figure 9, in 89% of the cases, the GPS was stopped in less than 20 s. For 92% of the samples, the process ended in less than 30 s, and for 97% of the samples, the process ended in less than 45 s. Only 1.7% of the GPS queries were finalized in more than 1 min.

These experimental data show the vast potential for optimizing GPS usage, comparing to a situation in which it is constantly enabled and searches for a position fix as long as possible. The data confirm our algorithm design presented in the previous subsection. As a more general conclusion, people spend the vast majority of their time stationary, as shown in [49], and briefly demonstrated by our Experiment 1 in the next section.

## 6. Sample Results

The experiments below demonstrate the capabilities of BX Tracker and evaluate its usefulness in the crowdsensing research. We present example data obtained by using our mobile application to show its versatility and potential in gathering mobility and network signal data. The data used in the experiments below were pre-processed to select adequate daily logs and merged into a single CSV file. Further filtering, analysis and basic visualization were performed in Microsoft Excel. We have measured the reference signal received power (RSRP) value reported by the LTE terminal (mobile phone or tablet) to represent the received signal level. The LTE signal strength heat map was generated using Google Maps, Python scripts, Gnuplot and Photoshop software. The density heat map of human mobility was created using Google Maps JavaScript API v3.

(1) Experiment 1: The first experiment analyzed a single user’s mobility during a five-day workweek and a two-day weekend collected on a smartphone. The data show the distinctive characteristics of human mobility, which were applied in [34] and described in [62]: repeated cyclic actions during the workweek and longer travels during the weekend. In seven days, the user traveled 27 km during the workweek and 191 km during the weekend. This gives characteristics similar to the Levy walk observed in [62]. Table 5 gives detailed information about the user activity during the whole measurement period.

**Table 5:** Mobility of a single user during one week (Experiment 1).

Activity	Duration	Percentage
Workweek		
still	114:21:53	94.19%
in-vehicle	1:00:00	0.82%
on-foot	6:03:09	4.98%
Weekend		
still	40:20:46	84.78%
in-vehicle	3:50:45	8.08%
on-foot	3:23:49	7.14%
Total weekly values		
still	154:42:39	91.54%
in-vehicle	4:50:45	2.87%
on-foot	9:26:58	5.59%

**Figure 10:**
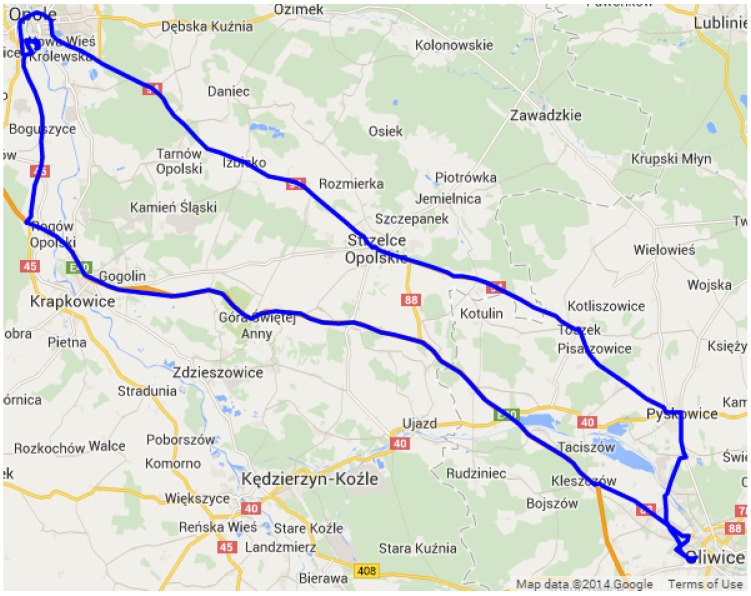
Daily GPS trace of a single user (Experiment 1). Map data ©2014 by Google.

An example of the user trace during a weekend trip is presented in Figure 10.

The data gathered using BX Tracker can be used for modeling various aspects of cellular networks; for example, discovering human mobility patterns, researching new mobility models [63], forecasting user behaviors in mobile networks [64], planning wireless network resource allocations [65], and more.

**Figure 11:**
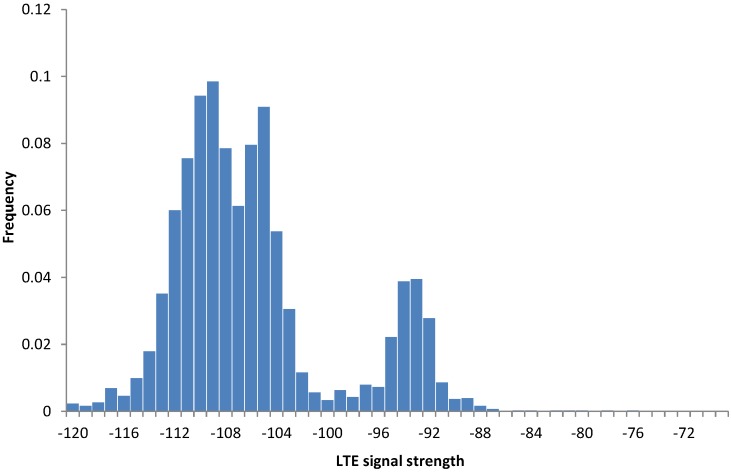
Signal strengths for all evaluated tablet devices (Experiment 2).

(2) Experiment 2: The experiment demonstrates local variations of the signal strength in an LTE network. We used three Asus ME302KL tablets equipped with mobile Internet SIM cards (registered in LTE Network 1) and one Samsung Galaxy S4 smartphone (registered in LTE Network 2). We were collecting the data for 18 days in the same office building. The data showed variations in the LTE signal strength, both for tablets and for the mobile phone. Figure 11 presents the signal level for all tablet devices in the same office building. The two significant peaks correspond to two nearby office rooms.

**Figure 12:**
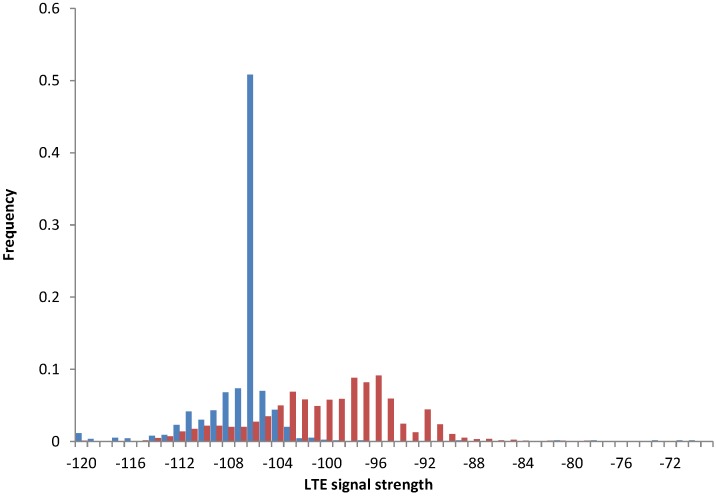
Comparison of signal strengths for a smartphone (blue) *vs*. a tablet (red) used in the same office room (Experiment 2).

Figure 12 presents the data collected on the smartphone (blue) and on a single tablet device (red), but placed in the same office spots. We observed substantial differences in stability of the signal, with the tablet signal resembling the normal distribution and the smartphone signal being much more stable.

**Figure 13:**
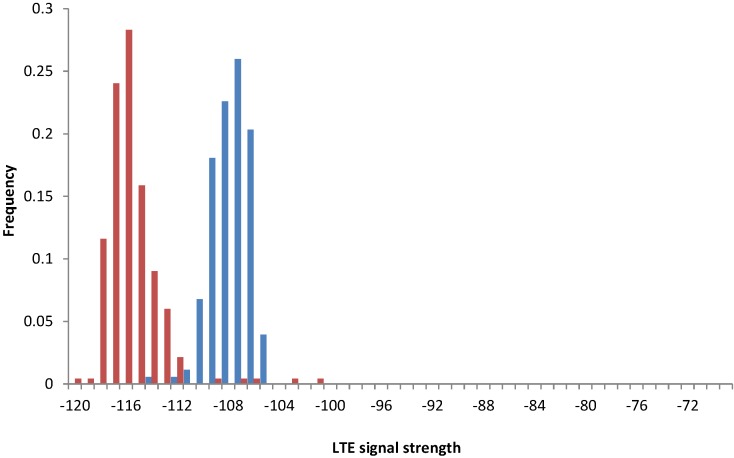
Signal strengths for two different locations in the same office room (Experiment 3).

**Figure 14:**
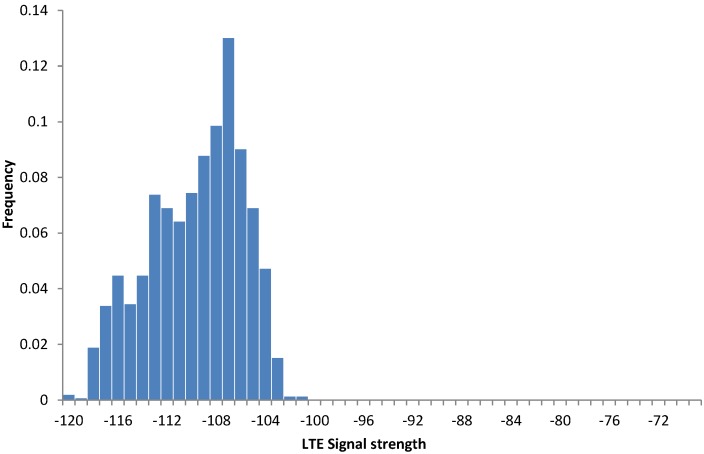
Signal strengths for 10 different locations in the same office room (Experiment 3).

**Figure 15:**
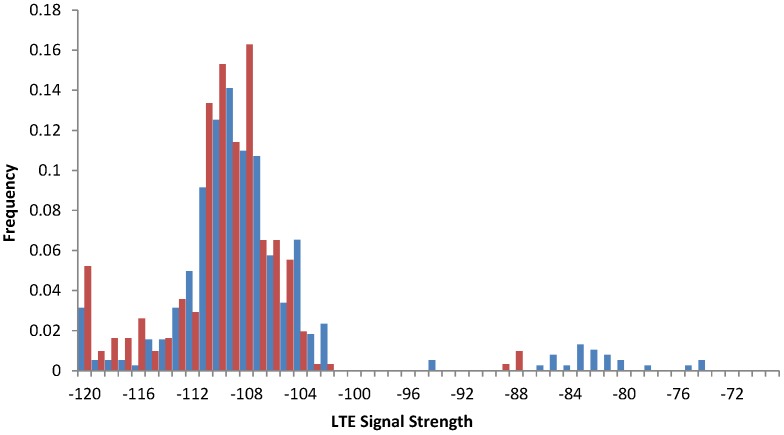
Signal strengths for movements on a single floor (blue) *vs*. many floors (red). (Experiment 5)

(3) Experiment 3: The experiment demonstrates variations in the LTE signal for a single office room. For 10 days, the same tablet device was placed for 5–6 h in different spots inside the same office room. Figure 13 shows the data for two different room spots, whereas Figure 14 shows the total signal variations for the whole experiment.

(4) Experiment 4: The experiment demonstrates variations in the LTE signal for a smartphone carried by an average office worker. The movement traces were collected for two days: the first day series involved traveling around many office rooms, but located on the same floor; the second day series involved many floors and the usage of an elevator. The data comparing both series are presented in Figure 15.

We calculated the time between connection technology changes (e.g., LTE to HSPA+); see Table 6. The main difference between the series is that in Series II, when the movement was more intensive, we can observe a lower percentage of LTE connections: it dropped from 78% down to 58%. However, the actual time between technology changes increased from 12 to almost 15 min.

**Figure 16:**
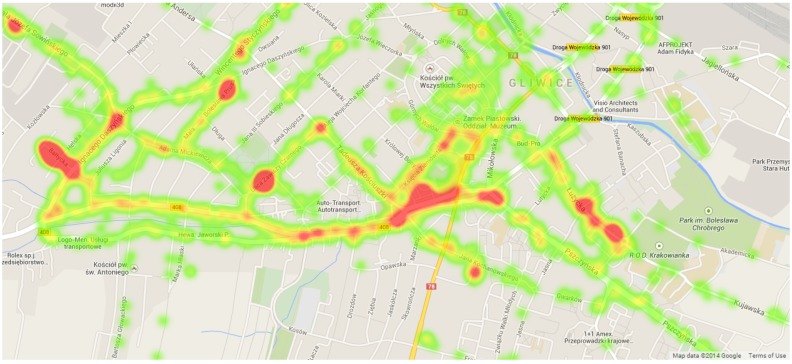
Density heat map of user locations (Experiment 5). Map data ©2014 by Google.

**Figure 17:**
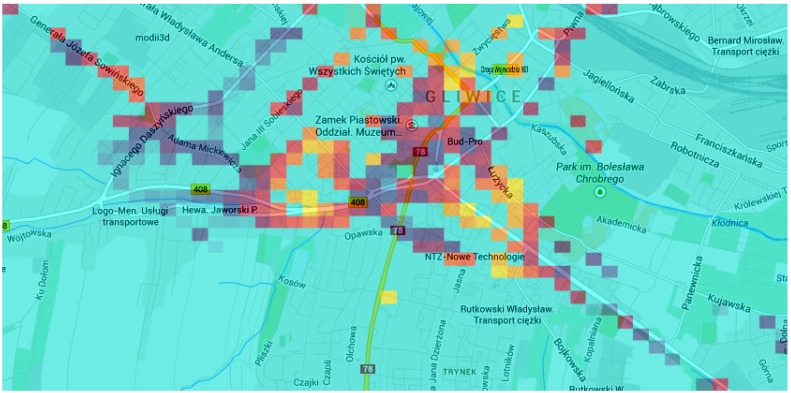
LTE signal heat map (map data ©2014 by Google).

(5) Experiment 5: The experiment visualizes popular locations of our test users for seven days in Gliwice, Poland. The heat map presented in Figure 16 has a few high density points, which correspond to work places, homes and other popular places, like shopping malls and restaurants.

(6) Experiment 6: The experiment visualizes real LTE signal strengths measured around the center of Gliwice. The resulting heat map is presented in Figure 17. The area is similar to the density heat map shown in Figure 16: we can observe that the highly popular places are not necessarily the areas with the best signal strengths.

**Table 6:** Dependence of connection technology on the movement pattern (Experiment 4).

Value	LTE	EDGE	HSPA	HSPA+
Series I: single floor				
Total time	03:37:44	00:04:02	00:40:57	00:15:09
Average time between changes	00:12:06	00:01:00	00:01:39	00:01:05
% of connections	78.36%	1.45%	15.04%	5.45%
Series II: many floors, elevator				
Total time	02:56:42	00:13:24	01:49:07	00:07:03
Average time between changes	00:14:43	00:03:21	00:04:36	00:00:53
% of connections	57.69%	4.38%	35.63%	2.30%
Total				
Total time	06:34:26	00:17:26	02:30:04	00:22:12
Average time between changes	00:13:25	00:02:11	00:03:07	00:00:59
% of connections	67.53%	2.98%	25.69%	3.80%

## 7. Conclusions

In this paper, a novel crowdsensing platform oriented towards minimization of the barriers limiting human engagement has been proposed. Our online survey confirmed that the battery drain and the requirement of interaction with the application are important aspects discouraging people from participation in a crowdsensing experiment carried through a smartphone application. We showed that these factors can be limited through the implementation of an energy-efficient GPS tracking and automatic detection of human activity by monitoring the accelerometer. The proposed BX Tracker application can gather information on the user location while reducing the device battery lifetime by only 20%. Moreover, 84% of our platform users experienced a GPS duty cycle of <5%, which corresponds to less than one hour per day. In comparison to traditional GPS tracking applications, the presented solution offers a 5% lower energy consumption, as is shown in Figure 7. This is achieved by enabling the GPS sensor only when the device is moving, by monitoring GPS satellite signal strengths to avoid using the GPS inside buildings and by adjusting the time pauses between successive GPS measurements.

We also presented various applications of the developed crowdsourcing platform in measuring human mobility and cellular signal levels. The results shown in Section 6 prove that BX Tracker can be used to efficiently collect data about the signal level distribution, maps of signal coverage and data about human mobility applicable to modeling cellular networks.
